# “*Hike up yer Skirt, and Quit.*” What Motivates and Supports Smoking Cessation in Builders and Renovators

**DOI:** 10.3390/ijerph10020623

**Published:** 2013-02-04

**Authors:** Susan J. Bondy, Kim L. Bercovitz

**Affiliations:** 1 Dalla Lana School of Public Health, University of Toronto, 155 College Street, Toronto, ON, M5T 3M7, Canada; 2 Ontario Tobacco Research Unit, 155 College Street, Toronto, ON, M5T 3M7, Canada; 3 The Research Doctor, Inc., Toronto, ON, (c/o Dalla Lana School of Public Health), Canada

**Keywords:** smoking cessation, workplace, qualitative research, blue-collar smokers

## Abstract

Construction-related occupations have very high smoking prevalence rates and are an identified priority population for efforts to promote cessation. This study sought to identify the smoking cessation supports and services which best suited this workforce group, and to identify gaps in reach of preventive health services. We performed qualitative text analysis on pre-existing conversations about smoking cessation among workers in this sector. The material appeared on a discussion forum about residential construction from 1998 and 2011. Roughly 250 unique user names appeared in these discussions. The qualitative analysis addressed knowledge, motivation, environmental influences, and positive and negative experiences with supports for cessation. Self-identified smokers tended to want to quit and described little social value in smoking. Actual quit attempts were attributed to aging and tangible changes in health and fitness. Peer-to-peer social support for cessation was evident. Advice given was to avoid cigarettes and smokers, to focus on personal skills, personal commitment, and the benefits of cessation (beyond the harms from smoking). Many discussants had received medical support for cessation, but behavioural counselling services appeared underutilized. Our findings support efforts toward more complete bans on workplace smoking and increased promotion of available behavioural support services among dispersed blue-collar workers.

## 1. Introduction

Tobacco use continues to represent a major source of preventable morbidity and mortality [1]. Smoking-related health consequences have been estimated to account for more than half of observed inequalities in health associated with diverse measures of socioeconomic status indicators, including occupation [2]. Recent surveys in Canada and the United States have shown smoking prevalence rates among blue-collar trades, and construction-related jobs, continue to be as high as thirty to forty percent and several times higher than the lowest rates observed by job type [3,4,5,6,7,8]. These observed rates show these specific worker populations to be a clear priority for tobacco control interventions.

There has been considerable research on tobacco use in many blue-collar settings, particularly where researchers have collaborated with large employers and unions [9,10,11,12,13]. However, the building and construction sector and has been more difficult to reach and study [14,15]. This sector presents unique challenges for health promotion research and programming because it is characterized by smaller companies, self-employed tradespersons, and dispersed job sites [16,17]. 

In recent decades, the gaps in smoking prevalence between high and low socioeconomic status (SES) groups have increased, largely because of earlier declines in smoking status among educated, higher SES and white-collar groups [18]. Earlier adoption of worksite tobacco control and cessation supports for white collar workers may have contributed to this inequity by job class [18,19]. The physical locations of construction work (often outdoors) have also often been excluded from worksite smoking bans [19,20,21,22]. As employer attitudes change (see [23]), inequality in smoking prevalence and burden could be further exacerbated if building tradespersons who smoke are displaced from the workforce, instead of helped to quit [23,24]. 

Therefore, public and private health organizations have identified construction-related occupations as a priority for increased delivery and use of programs to support smoking cessation [7,25]. However, little evaluative research exists to advise on the best models for this sector and governments have called for research to identify best models of smoking cessation programming for specific workplace settings including this sector [7,25,26,27,28]. These are also male-dominated work forces, for whom there is a need for research regarding the most effective treatment options [29]. 

The present study addresses these research gaps. We report on a qualitative analysis of conversations about smoking cessation, among workers in residential building and renovation, which appeared on the internet discussion board of a related trade magazine. Our previous report using qualitative analysis and this same data resource [23] focused on worksite smoking policies and produced rare insights into changing attitudes and tobacco-control practices on jobsites. It also provided key evidence that employers in this sector recognized the economic advantages of work-site smoking restrictions and of a smoke-free workforce [24,26]. 

This second study, using the same data resource, focused on the individual smoker’s motivations and experiences with cessation. Our research goals were pragmatic and sought to address two objectives. The first purpose of this study was to understand what motivates and assists smokers in this particular workforce to quit smoking. The second purpose was to capture the positive or negative experiences of these workers with diverse forms of cessation supports. 

## 2. Method

We performed qualitative analysis of existing text on an internet forum on residential building and design. The forum also served as a social network of individuals working in the construction and renovation sector. This resource included a unique volume of information about personal views and experiences with smoking and cessation, in the form of conversations which were neither solicited for the purpose of research nor restricted to people who had already volunteered for smoking treatment services (see also [23]). Study participants were all members of the Taunton Press “Breaktime” forum who engaged in conversations about smoking from 1998 through 2011 (roughly 250 unique board members). Because participants were not recruited for research, demographic data could not be obtained directly from participants (for further discussion about the sample and representativeness, see [23]). However, data from the publisher indicate that site users were mostly men (89%), working in residential construction and renovation in Canada and the United States, with an average age of 50, and higher incomes which are quite typical for this sector [23]. The researchers did not post messages or interact with board members as part of the research. The research was conducted with permission from Taunton Press, and ethical approval from the University of Toronto.

The data analysis approach was applied and descriptive and did not seek, explicitly, to advance theoretical perspectives on smoking or men’s health *per se* [30]. We sought to understand what these workers said motivated them to quit smoking. We also wanted to learn about the good and bad experiences these workers had had with programs and services for smoking cessation. In analyzing the data, we asked whether or not each subject heading (reflecting motivations, triggers to quit, factors that supported or were barriers to cessation, and methods used to quit and services used) was represented in the data. We then described the content of the material under each heading. 

A preliminary analysis framework was developed from multiple sources, starting with The Integrated Theory of Behavior Change (ITBC) developed in Nursing as a descriptive middle-range theory combining several theories of health behaviour [31]. Detail and breadth were added using sub-headings suggested by literature on smoking cessation, men’s health, and blue-collar smokers. Broadest headings included: knowledge, motivation, environmental and social influences, quit attempts, relapse and maintenance. We categorized content under motivation as reflecting either readiness or resistance to behaviour change [32,33]. Messages reflecting motivation were also classified as gain-framed (*i.e*., the good things that result from behaviour change) *versus* loss-framed (e.g., motivation to avoid illness or other adverse outcomes) [34]. When participants described personal experience with methods and services used to quit smoking, these were captured and classified as positive and negative experiences. One heading included masculine themes (e.g., social power, risk-taking, father, breadwinner), as some previous qualitative research on male, blue-collar smokers has suggested that a masculine social culture, or smoking as an emblem of masculinity, may represent unique barriers to cessation in male-dominated settings (e.g., [35]). We recorded the speaker’s self-described smoking status, which was usually offered [23]. 

Relevant text was found using searches for keywords: *smoke*, *smoking*, *cigarette(s)*, *tobacco*, *quit* and *stop smoking* as well as *cold turkey*, *nicotine*, *gum*, *patch*,and other cessation-related terms. Once found, entire threads were studied. Iterative searching stopped when new keywords and spelling variations produced no new content. To conduct the content analysis, entire posts were copied to word processing software under all applicable headings. Textual analysis software was not required for the volume of material (see [23]). Headings were expanded and collapsed based on heterogeneity of content. This was done, independently, by both authors who then discussed and reorganized the material until there was agreement. In presenting the results, key observations are presented organized in the approximate sequence: knowledge and motivation; attempts to quit; and maintenance of cessation. Headings with the most material are highlighted. We also comment on themes suggested in relevant literature, but for which there were few or no supporting data in this study. 

## 3. Results

Over 1,000 relevant messages were found, involving roughly 250 unique usernames. Messages were stereotypically brief, ranging from a few words to short paragraphs although some discussion threads about smoking and cessation extended over several years. Illustrative quotes for selected content areas are presented in Table 1. 

**Table 1:** Selected themes and illustrative quotations reflecting the described experiences of a community sample of builders and renovators with smoking cessation. Qualitative analysis of unsolicited conversations about smoking motivation, experiences and supports appearing on the Taunton Press internet forum “Breaktime” from 1998 to 2011.

Selected content areas and key observations	Selected, illustrative, quotations
**Motivation and cues to action**	
Knowledge of health effects evident Knowledge often linked to a general intention, but not necessarily to intentions to act, immediately, or quit attempts	*“I smoke, should quit, haven’t. Someday, someday yeah.”* *“Though her cause of death was health-related on several factors, it was never pinpointed to smoking, although I have no doubt it was a factor. I have dropped other bad habits over the years, but having a smoke is perhaps the hardest addiction to overcome. Time is fast approaching when I will reckon with this demon.”*
Cues to action included individual physician advice Age (middle age), often cited as reason for taking action	*“Doctor says I gotta quit now, so its time”* *“Two packs a day for 20+ yrs, am 44 now, want to see my kids mature.”*
**Barriers to trying to quit**	
Smoking described as enjoyable, and as an addiction	*“Frankly, I smoke because I enjoy it, like most, if not all, smokers.”* *“I aspire to be a non-smoker, and as much as you may think you know about it, if you have never been addicted to nicotine, you don’t have a clue as is how hard it is to quit.”*
**Role of social influences and social identity in continued smoking or cessation**	
Resistance to social pressures to quit	*“[…] the last way to get someone to quit smoking is to gripe about it all of the time.”* *“I lost a girlfriend once because she wanted me to quit and I wouldn’t; at least that was a lot of the reason.”*
**Reinforcement and barriers to success while trying to quit**	
Gain-based motivation and focus positive reinforcement of cessation	*“[…]. Man, I can’t believe the renewed energy I feel, and the sense of taste and smell is at an all time high for me. […].”* *“[…] Be better—better health, better tastin’ food, clearer mind, better sleeping habits….and you will never smell like smoke to your friends, relatives and customers. Sit through a two hour movie with your love […] travel in someone else’s car whenever you want.”* *“We go more places and we like the people better.”* *“I started taking flight lessons. Had to give up cigarettes because I couldn’t afford them anymore.”*
Messages of encouragement emphasizing personal commitment and self-regulation	*“[…] the only way you can quit is if you truly WANT to. For whatever reason. Doesn’t matter what motivates the desire. If you truly want to, you will.”* *“If you cannot see others’ reasons and lessons and nagging to stop, then you need the reason in yourself.”* *“People find it hard psychologically to quit when they think they will never smoke a cigarette again and that this puff is to be their last. It’s tough! Instead, don’t think you have to quit forever, but rather you choose not to smoke at this time.”* *“Simply take it day by day and those days will turn into weeks and the weeks into months and hopefully years. […] Just do them one at a time, easy does it.”*
Advice on relapse avoidance focusing on the physical environment, access to cigarettes and presence of smokers	*“Sure would be easy to bum a smoke or two and become a regular smoker again. I don’t mind having sporadic contact with smokers, just can’t work right next to one.”* *“Just stop buying them. You start to quit when you stop buying them. You know you’ve got them beat the day someone flips a cigarette out a car window and you don’t stop your car and chase after it.”* *“So I don’t [smoke at work] mostly because of the company policy. A little for the non smokers, and largely for the few who are trying to make a go of quitting; partially or completely.”*
Peer-to-peer support sought and offered	*“Well I have finally come to the point that I WANT to quit smoking, not to mention the fact that I need to. Open to suggestions from reformed smokers, […]. “* *“Good Luck with whatever you try, and let me how it goes. Maybe I’ll finally try to kick the habit.”* *“Speaking of smoking […], how’s the quitting going? Been pulling for you.”*
**Experiences with cessation strategies, interventions and supports**	
Pharmacotherapy and health professional support most evident.	*“The trick is to make sure you use the right strength of patch and to go the full month at each strength; also take the patch off before bedtime […] the nicotine does weird things to your brain/body when you’re trying to rest. Another important thing is take the patch off before exercise/activity—this includes any form of exertion (see: take the patch off before bedtime). […]”* *“Talk to a doctor and try zyban. I’ve been smoke free for 1 year 4 months and I feel great. Zyban helped, but this stuff sort of makes you restless.”*
Recommendations to combine physical fitness with smoking cessation.	*“…Get a gym membership. […] Three times a week […] and I’ve never felt better in my life! The added bonus is that it revs the metabolism, so that when you get the munchies (as a smoke substitution), you’re burning them off and don’t get the post-quit-poundage.”*
Strategy advice included warnings against cutting back; mixed views on need for medication.	*“Stop completely! No more at all. Don’t cut back just stop. I know I tried that a couple of times.”* *“Cutting down never works…of all my friends who have quit, the ones who did it cold turkey are still non-smokers today.”* *“I respect anyone who can do it cold turkey but many need some help in slowly getting off the nicotine.”*

As described in our previous report which focused on workplace smoking policies [23], speakers tended to be aware of smoking-related illness, and described themselves as knowing they “should” quit. However, it was common to report only a general intention to quit at an undefined future date. Experiences which were described as leading to actual behaviour change included self-perceived changes in health, energy or fitness, and specific health advice from a physician. Several speakers cited their age, *per se*, or age-associated increases in health risk, as the reason for their decision to act or seek advice on how to quit smoking. 

Where positive motivation to continue smoking was described, it was in terms of enjoyment (see Table 1) and to avoid adverse physical effects of quitting. Many smokers described themselves as addicted. 

In terms of social influences, smokers said they expected others to disapprove of smoking and see it as *repulsive*, or *smelly.* Several described feeling harassed about their smoking (see [23]) but no speakers stated if this harassment increased motivation to quit or decreased it (e.g., as a result of stress). Some smokers reported showing resistance to direct pressure to quit or stated that pressure to quit from close personal contacts had been ineffective (See Table 1). Smokers often stated that quitting had to be a personal goal or it would not happen. Others emphasized autonomy and encouraged others to quit smoking “*for themselves*”. Similarly, statements which most closely matched the concept of social reinforcement of continuing to smoke consisted only of descriptions of high smoking prevalence or smoking breaks. There were no statements which demonstrated that smoking played an important role in continuing or enhancing inter-personal relationships. Speakers did identify specific social settings (e.g., bars) as presenting a risk for relapse, but when any reasons for this were given, the speakers described only physical access to cigarettes, and the smell as a trigger to smoke. 

In terms of masculinity, as a theme area, continued smoking was described negatively in terms of being the bread-winner (see also [23]), father or grandfather. This was expressed in terms of being around to see children mature, and protecting children. Under the sub-heading of smoking as a sign of social power or masculine strength, we previously reported [23] that workplace authority figures were, increasingly over time, expected to take a strong stand against smoking. We also found statements (arguably) masculine in tone (*i.e*., assertive, curt, with cursing or capital letters, or using aggressive humour) promoting the rights of both smokers and non-smokers alike. In this analysis, we also noted jokes about impotence from smoking, and this humorous note of encouragement in a conversation about the fortitude required to quit: “*Hike up yer skirt, Mary! And stop!*”

Participants asked for and offered advice on quitting and described methods they had used. Messages of success, and encouragement from former smokers to peers, were predominantly framed as gain-based [34]. The benefits of cessation cited most often were: improved fitness; social benefits, entertainment and recreation, and cost-savings. Financial benefits were reported as gain-based. Quitters and former smokers emphasized increased discretionary income for leisure activities and luxuries. We also found numerous strong recommendations to start fitness campaigns as a direct aid to cessation, and to reward to oneself for quitting. 

Most threads describing formal cessation treatment or support described the use of stop-smoking medications approved and recommended for smoking cessation in the United States and Canada: nicotine replacement therapy (NRT) in gum and patch form; bupropion; and, varenicline [36,37,38,39]. While several speakers asked about the effectiveness of herbal or alternative therapies, in all these discussion threads, messages appeared which recommended seeking medical advice or similar scientific evidence of effectiveness. Almost all messages which described support provided by a health professional or counselor referred to physicians. 

In contrast to the volume of discussion about medication, no speaker described having used behavioural interventions or supports (e.g., print materials, quit lines, or support groups). We found just one message with a clear reference to behavioural counselling; however, the speaker referred to 12-step addictions programs, generally, and neither self-identified as a smoker nor stated that this was personal experience. 

Despite there being no described use of support groups, peer-to-peer social support was very much in evidence (see Table 1). Help and advice on how to quit was both sought and offered. The content of these exchanges included personal motivation, behavioural strategies, and self-regulation skills, all of which are consistent with cognitive and behavioural counselling methods for smoking cessation. 

Former smokers commented on the strategies they used to quit. There were several exchanges about quitting “*cold turkey*” meaning, from context, quitting without medication. Some smokers recommended medication; others emphasized that willpower and personal commitment were more important. Some speakers used the term “*cold turkey*” meaning to quit completely, *versus* cutting down. In these conversations, strongly-worded advice was given by several speakers to not try to cut back but to quit completely (Table 1). 

A very common discussion topic was the expected duration of withdrawal symptoms and relapse risk. These could last many years. Some smokers and successful quitters described relapse without any conscious intention to smoke. Where advice was offered on how to avoid relapse, it was to avoid cigarettes and physical cues (e.g., ashtrays, smell). 

## 4. Discussion

This unique resource [23] previously showed that smoking attitudes and policies were changing in building and renovation workplaces. The present analysis focused on the individual builder and factors that motivate and help him to quit. We observed considerable interest in smoking cessation and peer-to-peer social support. We also found high levels of awareness of the health effects of smoking and stop-smoking medication, but little use of behavioural counselling or support groups. Our analysis refutes suggestions that builders have uniform pro-smoking attitudes, or that smoking is always part of their social identity. Our findings are more consistent with those of Katainen, *et al.* [40] who described smoking among blue-collar smokers as “*an individual habit without any shared symbolism*” (p. 1092). Most discussants knew they should quit and wanted to. They simply found it hard to do. 

An acknowledged limitation of this research (see also [23]) is potential lack of representativeness of the sample, in a setting where most of the available literature is based on convenience samples and individual workplaces [10,28]. We cannot claim that our findings reflect all workers in residential building and design, workers in other settings, or those with lower incomes or computer literacy. Our study population cannot be compared directly to other studies of research volunteers. Workers who are more likely to adopt healthy behaviours may also be more likely to participate in workplace health research [41]. Another limitation is that we cannot know if speakers used, but never mentioned specific cessation services, although this seems unlikely. It is a limitation of this study design in that this could not be asked directly. Complementary designs such as quantitative surveys are also needed. 

This research has several implications for health promotion messages to motivate cessation and promote use of cessation services. We suggest the use of an up-beat, deliberately humorous tone, which emphasizes the positive benefits of cessation. Negative messages, references to social norms, and nagging should be avoided. Researchers in many settings encourage use of gain-framed motivational messages [42,43,44,45,46] and our findings support this. Messages which focus mostly on adverse health effects are, arguably, a low priority. Messages which emphasize achievable health benefits may be more persuasive. Smoking cessation may be combined with other positive behaviour changes to improve health overall [47]. We also suggest that programs for this target audience appeal to intrinsic motivation, individualism, and personal autonomy. This is consistent with some observations in a recent systematic review of studies of workplace-based smoking cessation programs for the British Occupational Health Research Foundation [28]. Positive and intrinsic motivation was more important than external incentives [28]. Wanting to gain autonomy over smoking has been cited as a key motivation in many smoker populations [48].

Many of these smokers took action to quit because of reaching middle-age. This matches the age distributions of smokers using cessation clinics [37,49] and quit-lines [50]. It is interesting to note that interventions for blue-collar smokers designed for any specific age group are most likely intended for young workers entering the workforce (for examples, see [51]). Programs should also address the motivations and needs of older smokers. While nobody recommends waiting to quit, quitting even in middle age greatly reduces the years of life lost due to smoking [52,53]. Any intervention which gets more smokers to quit can also reduce the number of active smokers in the workplace, and so also help protect younger workers from pro-smoking influences and cigarette smoke exposure at work [54,55]. 

In terms of experience with cessation services, physician intervention [49] was best known and did lead to behaviour change. Comments that the perception of health risk had to be concrete and personal, is consistent with the goals of intervention models which use personalized health risk assessment [56]. However, there is insufficient evidence to show this technique increases the likelihood of quitting [56]. It may be that unprompted recognition of health changes are most important (see also [57]). 

For many smokers trying to quit, medications were seen as helpful. Medications have proven benefit for men and blue-collar workers in controlled treatment settings [29]. In contrast to the frequency with which medications were discussed, behavioural interventions appeared to be greatly under-used, and should be promoted. Well-established behavioural intervention models include brief interventions through intensive individual models such as motivational interviewing designed to address ambiguity in intention and focus attention on the positive aspects of cessation [58.59]. These approaches are effective when delivered individually or in group sessions, face to face or by phone [58,59]. These programs require trained staff, but can be cost effective relative to the costs of smoking [60,61]. Costs can be reduced by using the internet and telephone [63]. Campaigns at work to promote counselling services increase quit attempts, and are effective in blue-collar populations [12,63,64]. The challenge, then, is how to promote use of these supports by workers in distributed work-places.

Here, the underuse of behavioural interventions was not surprising as construction workers are often self-employed or work in small companies without workplace-based health promotion services or supplemental insurance [65,66]. However, over the time period of the study, telephone and web-based cessation services became available to the general public in Canada and the United States and would have been available regardless of insurance [8]. Further effort is warranted to increase awareness of these services. These are an important adjunct to physician advice, in Canada, where most behavioural treatment is delivered by non-physician providers [7], and in the United States where private and public health insurance coverage for cessation services are variable [62]. 

At least some of these (mostly male) builders and renovators were willing to engage in mutual support over the internet with acquaintances and anonymous peers. Blue-collar smokers may be receptive to support groups involving people with the same occupation or interests. A previous focus group study reported that peer support in cessation from co-workers was valued, but that it was better to not mix different classes of workers [28]. Future research should ask whether blue-collar and male workers prefer support groups which are more anonymous, and involve people like themselves. Carefully developed partnerships with private businesses (whose clientele share demographic characteristics, or interests) may be a way to promote cessation services, and develop support groups. 

These workers also commented on specific techniques to quit which are the subjects of ongoing research. Exercise has long been recommended in smoking cessation [67,68] and increases rates of abstinence at least when the exercise is maintained [12,67,68]. Further research should determine if combined smoking and exercise programs are particularly helpful for individuals who do physical work and may be more sensitive to physical changes. 

Several of our study subjects commented on cutting down. There is active research in the use of staged cessation (cutting down first) to increase cessation rates in heavy smokers [69]. Several of our speakers gave strong advice not to quit completely, feeling strongly and that trying to cut back would not work. Naturally, this non-intervention study provides no direct evidence of the effectiveness of the methods discussed by speakers. 

Finally, the research points to the expansion of worksite smoking bans to help blue-collar workers quit and maintain abstinence. Workplace restrictions have reduced smoking prevalence in many but not in all blue-collar work settings [13,63,70]. Workplace bans have often exempted outdoor areas, and structures being built or demolished (see also [23]). Whole property bans are feasible. In many North American jurisdictions entire-site smoking bans are required with building or demolition permits, but may not be enforced. Examples of bans which are enforced include specific municipalities (e.g., New York City) or bans enacted by property owners or employers. 

In conclusion, our study suggests that many builders who smoke want to quit and are aware that medications can help. However, behavioural interventions and support groups were underused and should be promoted. Our study also suggests that blue-collar smokers may benefit from social support from true peers, when trying to quit, and that the internet may be an effective way to create support networks among dispersed groups of blue-collar smokers. 
